# Mixed Infant Feeding Is Not Associated With Increased Risk of Decelerated Growth Among WIC-Participating Children in Southern California

**DOI:** 10.3389/fnut.2021.723501

**Published:** 2021-10-28

**Authors:** Christopher E. Anderson, Shannon E. Whaley, Catherine M. Crespi, May C. Wang, M. Pia Chaparro

**Affiliations:** ^1^Division of Research and Evaluation, Public Health Foundation Enterprises WIC, Irwindale, CA, United States; ^2^Department of Biostatistics, Fielding School of Public Health, University of California, Los Angeles, Los Angeles, CA, United States; ^3^Department of Community Health Sciences, Fielding School of Public Health, University of California, Los Angeles, Los Angeles, CA, United States; ^4^Department of Social, Behavioral, and Population Sciences, School of Public Health and Tropical Medicine, Tulane University, New Orleans, LA, United States

**Keywords:** infant formula, breastfeeding, WIC, growth faltering, child growth

## Abstract

**Background:** The Special Supplemental Nutrition Program for Women, Infants, and Children (WIC) provides nutrition assistance to half of infants born in the United States. The nationally representative WIC Infants and Toddler Feeding Practices Study-2 (ITFPS-2) reported a caloric deficit at 7 months among infants receiving WIC mixed feeding packages, suggesting these infants may be at risk for growth deceleration/faltering.

**Methods:** Longitudinal administrative data collected prospectively from WIC participants in Southern California between 2010 and 2019 were used (*n* = 16,255). Infant lengths and weights were used to calculate weight-for-length (WLZ), weight-for-age (WAZ) and length-for-age (LAZ) z-scores at different time points. Growth deceleration/faltering was determined at 9, 12, 18, and 24 months by the change in z-score from the last measurement taken ≤ 6 months of age. Infant feeding was categorized by the food package (breastfeeding, mixed feeding, and formula feeding) infants received from WIC at 7 months. Poisson regression models were used to evaluate the association between WIC infant package at 7 months and deceleration/faltering at 9, 12, 18, and 24 months.

**Results:** The proportion of infants displaying decelerated/faltering growth was low for all infant food package groups. Receiving the WIC mixed feeding package at 7 months of age was not associated with WLZ, WAZ, and LAZ deceleration/faltering growth.

**Conclusions:** Growth deceleration/faltering rates were very low among WIC participating children in Southern California, highlighting the critical role of nutrition assistance in supporting adequate growth in early childhood.

## Introduction

The first 1,000 days of a child's life represent a critical period, with adequate nutrition imperative for healthy growth and development (1). The American Academy of Pediatrics (AAP) has recommended exclusive breastfeeding from birth to 6 months of age, and continued breastfeeding with the appropriate introduction of complementary foods and beverages (2) until 1 year of age (3). The AAP Committee on Nutrition has contended that breastmilk, with appropriate complementary foods, is nutritionally optimal for infants (1). Rates of breastfeeding initiation and through 6 months of age remain below the benchmarks set in Healthy People 2020 (4), and are lower for infants living in low-income households (5).

The Special Supplemental Nutrition Program for Women, Infants and Children (WIC) is a nutrition assistance program of the federal government of the United States and is administered by the United States Department of Agriculture, serving over 6.2 million participants monthly in 2020 (6). WIC provides supplemental foods to eligible mothers and children in households with incomes below 185% of the federal poverty level (FPL), in addition to nutrition education, breastfeeding promotion and social/medical service referrals (7). For infants, food packages are tailored based on the amount of breastfeeding reported by their mothers, with infant formula provided to supplement breastmilk for infants who are not exclusively breastfed, or as the main source of feeding for those exclusively formula fed (8). WIC food packages were modified in 2009 to align them with dietary guidelines, promote breastfeeding (9), and address high rates of childhood obesity (10). Improvements in diet quality among WIC-participating families (11), increased rates of exclusive breastfeeding and breastfeeding duration (12) and decreased obesity rates (13) have been reported.

The nationally-representative WIC Infant Toddler Feeding Practices Study-2 (ITFPS-2) reported a substantial caloric intake deficit at 7 months of age among infants receiving mixed feeding packages, compared to infants receiving the fully breastfeeding or fully formula feeding packages (14). The reported caloric deficit among infants receiving mixed feeding packages suggest that mothers who continue to partially breastfeed beyond the introduction of complementary feeding, recommended around 6 months of age (3), may experience difficulty in meeting the nutritional needs of their infants, compromising child growth and undermining the benefits of nutrition assistance. However, the design of ITFPS-2 did not allow for an evaluation of the growth of those children experiencing a caloric deficit. The objectives of this study were to determine the percent of WIC-participating children who experienced growth deceleration/faltering after 6 months of age, and to assess the relationship between the food package received at 7 months of age and growth deceleration/faltering. Based on the findings of ITFPS-2, we hypothesized that growth deceleration/faltering would occur in a higher proportion of infants who received mixed feeding WIC food packages at 7 months, compared to those who received the formula feeding or breastfeeding WIC infant packages at 7 months of age.

## Methods

### Subjects

Administrative data collected in Southern California between 2010 and 2019 by Public Health Foundation Enterprises (PHFE) WIC, the largest WIC local agency in the United States, were used in this study. Children were included in the analysis if they: were enrolled in WIC continuously from birth (within 42 days) to after 1 year of age, had complete WIC infant package data for 12 or more of the first 13 months of life; had at least two length and weight measurements separated by more than 3 months before 6 months of age and at least one length (or height) and weight measurement between 7 and 26 months of age, and; had complete sociodemographic covariate data (child sex and race/ethnicity; maternal language preference and education; household income). Infants born pre-term, with developmental delays, or with genetic or congenital conditions that may affect feeding or metabolism and consequently growth, including Down syndrome and cleft palate, were excluded from the sample.

### Independent Variables

The WIC food package received at 7 months of age was the independent variable of interest in this study. It differed according to whether the infant was fully breastfed (“breastfeeding” package), partially breastfed (“mixed feeding” package), or fully formula-fed (“formula feeding” package). The breastfeeding package did not include infant formula; the mixed feeding package included some formula, with quantities varying based on the amount of breastfeeding reported; and the formula feeding package contained enough formula to meet the infant's age-specific nutritional needs, taking into account nutritional needs expected to be met by complementary foods beginning at 6 months of age (9).

Covariates available from WIC administrative data include the child's age, birth year, sex, and caregiver-reported race/ethnicity (Asian, Black, Hispanic, non-Hispanic White, or Other); maternal education (less than high school, completed high school, or more than high school) and preferred language (English, Spanish, or other language); and household income (categorized as <50% FPL, 50–100% FPL, and >100% FPL).

### Outcome

Growth deceleration/faltering was the outcome of interest and was assessed using length (or height) and weight measurements collected by WIC during service delivery (i.e., certification and recertification). Anthropometric indices- length-for-age (LAZ), weight-for-age (WAZ), and weight-for-length (WLZ)- were calculated from these measurements taken from birth to <39 months of age, using sex-specific CDC growth curves (15, 16). As recommended by the United Kingdom National Institute for Health and Care Excellence (17), these indices were categorized into 10 groups based on their corresponding percentiles: <0.4th, 0.4th to <2nd, 2nd to <9th, 9th to <25th, 25th to <50th, 50th to <75th, 75th to <91st, 91st to <98th, 98th to <99.6th, and >99.6th. Changes in categories between the last measurement at ≤ 6 months of age and each measurement after 7 months of age through the last measurement were used to identify children who exhibited decelerated/faltering growth in late infancy. Similar guidelines from the US are not available. Growth deceleration/faltering occurred when the follow-up measurement for a specified anthropometric index was below the 9th percentile and the index decreased (1) ≥1-category if below the 9th percentile at ≤ 6 months; (2) ≥2-categories if below the 91st percentile at ≤ 6 months; or (3) ≥3-categories for any percentile value at ≤ 6 months.

To focus on the impact of the WIC infant food package received at 7 months on growth deceleration/faltering, infants who showed evidence of poor growth during the first 6 months of life, namely infants with any anthropometric index <2nd percentile or who showed a decrease in any anthropometric index percentile category between two measurements at ≤ 6 months of age, were excluded (18). The selection of the final analytic sample (*n* = 16,255) is shown in the Supplementary Figure 1.

### Statistical Analysis

The characteristics of children receiving breastfeeding, mixed, or formula packages from WIC at 7 months of age were described using means and standard deviations or frequencies and percentages, as appropriate. The associations between the WIC infant package received at 7 months and growth deceleration/faltering were evaluated in modified Poisson regression models with robust standard error estimation, which were used to calculate adjusted risk ratio (RR) estimates accommodating repeated measurements for children (19, 20). The models were adjusted for household income, maternal education and language preference, initial anthropometric index percentile category, sex, and race/ethnicity. The models used polynomials for age at measurement (linear, quadratic, and cubic) and interaction terms between age polynomial terms and the WIC package received at 7 months of age to allow the associations between infant package and growth deceleration/faltering to vary non-monotonically with age. Model fit was compared between models removing the highest order age polynomials sequentially and interactions between the highest order polynomial and WIC package received at 7 months; model fit was compared with the quasi-likelihood information criterion. Model fit and estimated associations were similar between models with the quadratic and cubic polynomials as the highest order age variable. The model with the cubic term was retained as the final model to allow for less constrained (i.e., non-symmetrical) variation in the relationship between WIC infant package and growth deceleration/faltering across the observed age range. Parameter estimates from the final models are presented in the Supplementary Table 1. *P*-values < 0.05 were considered statistically significant. All analyses were conducted using SAS 9.4 (SAS Institute Inc., Cary, NC).

## Results

The analysis was conducted on 16,255 children with 116,398 unique length (or height) and weight measurements. At 7 months of age, 17.0% of children received the breastfeeding package, 22.6% of children received the mixed feeding package, and 60.3% of children received the formula feeding package. Most children in each infant package type were Hispanic (Table 1). Children receiving the formula feeding package at 7 months of age were more likely to have a mother who preferred to speak English and live in a household with income below 50% FPL than children receiving mixed feeding and breastfeeding infant packages at 7 months of age.

**Table 1:** Characteristics of WIC-participating children in Southern California, 2009-2019, by WIC infant package received at 7 months (*N* = 16,255).

	**WIC infant package received at 7 months of age**		
	**Formula feeding**	**Mixed feeding**	**Breastfeeding**
**Characteristics**	***N* = 9,805**	***N* = 3,680**	***N* = 2,770**
Initial WLZ^a^, mean ± SD	0.94 ± 0.89	0.97 ± 0.89	1.06 ± 0.91
Initial WAZ^a^, mean ± SD	1.01 ± 0.91	1.02 ± 0.91	1.11 ± 0.96
Initial LAZ^a^, mean ± SD	0.46 ± 0.8	0.44 ± 0.77	0.45 ± 0.78
Age (y) at initial WLZ, mean ± SD	0.46 ± 0.02	0.46 ± 0.02	0.46 ± 0.02
Age (y) at last WLZ, mean ± SD	2.51 ± 1.36	2.73 ± 1.39	2.72 ± 1.38
Male, *n* (%)	5,045 (51.45)	1,905 (51.77)	1,375 (49.64)
**Race/ethnicity**, ***n*** **(%)**			
Asian	686 (7.00)	422 (11.47)	187 (6.75)
Black	459 (4.68)	105 (2.85)	91 (3.29)
Hispanic	8,278 (84.43)	2,983 (81.06)	2,352 (84.91)
Non-hispanic white	194 (1.98)	101 (2.74)	94 (3.39)
Other	188 (1.92)	69 (1.88)	46 (1.66)
**Maternal language**, ***n*** **(%)**			
English	6,744 (68.78)	1,632 (44.35)	1,463 (52.82)
Spanish	2,564 (26.15)	1,674 (45.49)	1,158 (41.81)
Other	497 (5.07)	374 (10.16)	149 (5.38)
**Maternal education**, ***n*** **(%)**			
< High school	3,998 (40.78)	1,648 (44.78)	1,070 (38.63)
High school graduate	4,066 (41.47)	1,222 (33.21)	1,038 (37.47)
>High school	1,741 (17.76)	810 (22.01)	662 (23.90)
**Household income**, ***n*** **(%)**			
<50% FPL	3,125 (31.87)	823 (22.36)	590 (21.30)
50–100% FPL	4,180 (42.63)	1,791 (48.67)	1,309 (47.26)
>100% FPL	2,500 (25.50)	1,066 (28.97)	871 (31.44)

*FPL, federal poverty level; SD, standard deviation; LAZ, length-for-age z-score; WAZ, weight-for-age z-score; WLZ, weight-for-length z-score; WIC, Special Supplemental Nutrition Program for Women, Infants and Children; y, years*.

a *Initial WLZ, WAZ, and LAZ are based on the CDC growth standard corresponding to the baseline measurement of length and weight in the dataset for each subject (i.e., the last measurement before 0.5 years of age)*.

**Figure 1:**
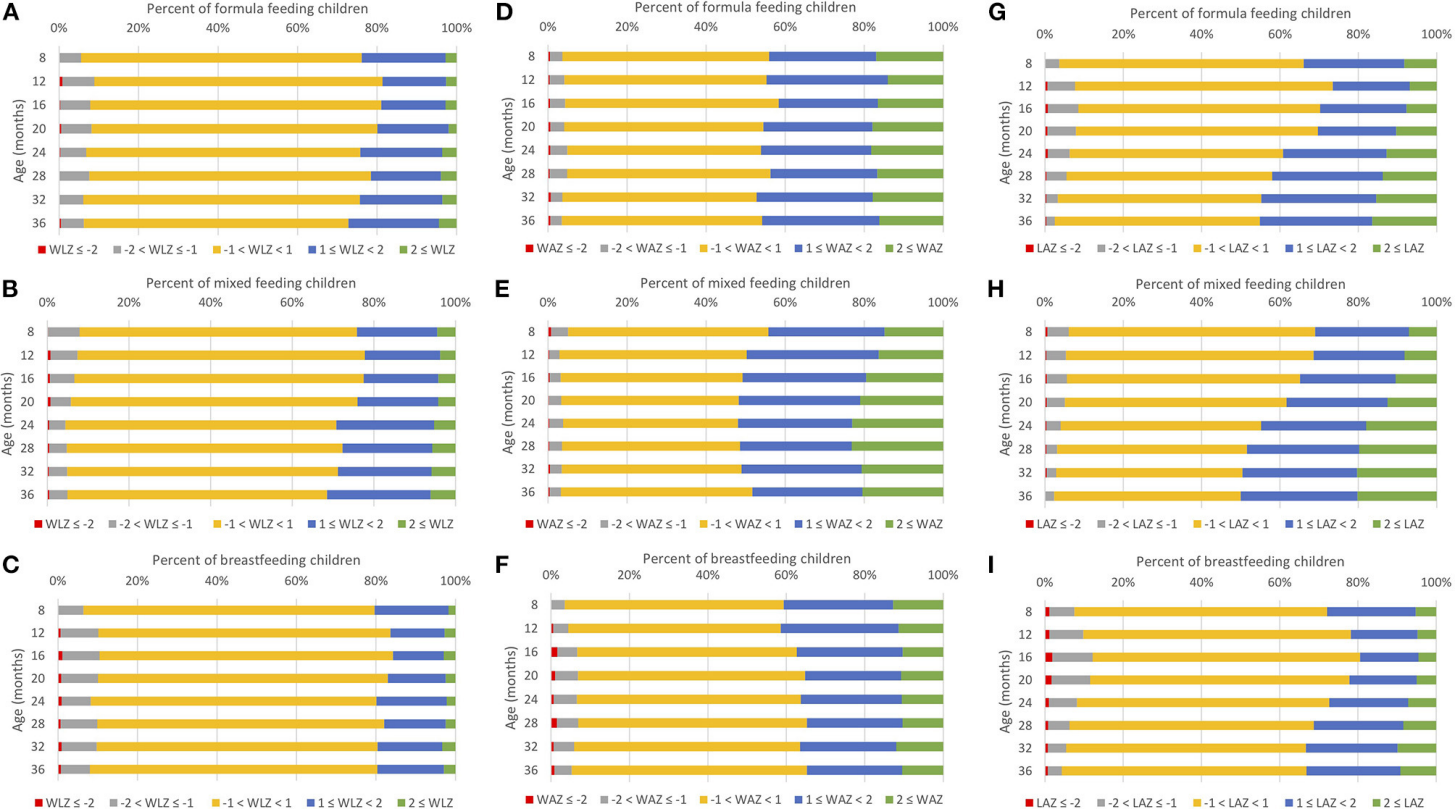
Prevalence of anthropometric index percentile categories^a^ by infant package received at 7 months for WIC-participating children from ages 7 to 38 months in Los Angeles County, 2009–2016 (*N* = 16,255). **(A)** Formula feeding, WLZ, **(B)** Mixed feeding, WLZ, **(C)** Breastfeeding, WLZ, **(D)** Formula feeding, WAZ, **(E)** Mixed feeding, WAZ, **(F)** Breastfeeding, WAZ, **(G)** Formula feeding, LAZ, **(H)** Mixed feeding, LAZ, **(I)** Breastfeeding, LAZ. ^a^Categories were defined by z-scores for each anthropometric index: z ≤ 2, −2 < z ≤ −1, −1 < z <1, 1 ≤ z <2, 2 ≤ z. Age was categorized: 7 to <10 m (8 months), 10 to <14 (12 months), 14 to <18 (16 months), 18 to <22 (20 months), 22 to <26 (24 months), 26 to <30 (28 months), 30 to <34 (32 months), 34 to <39 (36 months). LAZ, length-for-age z-score; WAZ, weight-for-age z-score; WLZ, weight-for-length z-score.

Across all ages of follow-up in this study (7 to <39 months), and for all infant package groups, a large majority of children demonstrated normal (−1 < z-score <1), high (1 ≤ z-score < 2) or very-high (2 ≤ z-score) values for all anthropometric indices (Figure 1). A smaller proportion of children who received the breastfeeding package at 7 months were in high and very-high categories for WLZ, WAZ, and LAZ (Figures 1C,F,I, respectively) than children who received the mixed feeding package (Figures 1B,E,H) or the formula feeding package (Figures 1A,D,G). The higher proportion of normal values among children who received the breastfeeding package was particularly evident for WLZ (Figure 1C) and WAZ (Figure 1F). A modestly larger proportion of children who received the breastfeeding package demonstrated low (−2 < z-score ≤ −1) and very-low (z-score ≤ −2) index values than children who received mixed feeding and formula feeding packages.

Decelerated/faltering growth between ≤ 6 months of age and 7 to <10, 10 to <14, 14 to <18, 18 to <22, and 22 to <26 months of age was observed among 1.1–3.3% of children, depending on the age range and anthropometric index: for WLZ = 1.5, 1.3, 1.8, 1.9, and 2.0% in each age range, respectively; WAZ = 1.1, 2.3, 3.3, 3.0, and 2.4% in each age range, respectively; and LAZ = 1.5, 2.8, 2.6, 2.3, and 1.9% in each age range, respectively. A majority of growth deceleration/faltering for each anthropometric index was observed among children who were between the 25th and 75th percentile at ≤ 6 months of age (Table 2).

**Table 2:** Frequency of growth deceleration/faltering^a^ between 6 months and ages up to 26 months among WIC participating children in Southern California, by initial CDC anthropometric percentiles (*N* = 16,255).

	**Growth deceleration/faltering by age (months)**				
	**7 to <10**	**10 to <14**	**14 to <18**	**18 to <22**	**22 to <26**
	***N*^b^ = 883**	***N*^b^ = 15,332**	***N*^b^ = 5,291**	***N*^b^ = 6,436**	***N*^b^ = 9,082**
	**WLZ**				
**Baseline percentile**	***N***	***N***	***N***	***N***	***N***
Any, *n* (% of total)	13 (1.5)	205 (1.3)	93 (1.8)	120 (1.9)	186 (2.0)
2 to <9	3	4	3	4	2
9 to <25	1	8	6	4	6
25 to <50	3	82	39	39	57
50 to <75	5	68	23	48	65
75 to <91	1	32	13	18	44
≥91	0	11	9	7	12
**Baseline percentile**	**WAZ**				
Any, *n* (% of total)	10 (1.1)	350 (2.3)	175 (3.3)	193 (3.0)	214 (2.4)
2 to <9	2	8	2	2	1
9 to <25	1	30	13	15	16
25 to <50	7	201	85	83	101
50 to <75	0	85	63	69	61
75 to <91	0	15	9	15	24
≥91	0	11	3	9	11
**Baseline percentile**	**LAZ**				
Any, *n* (% of total)	13 (1.5)	429 (2.8)	136 (2.6)	151 (2.3)	174 (1.9)
2 to <9	0	11	3	4	1
9 to <25	1	43	12	18	12
25 to <50	8	230	73	78	97
50 to <75	3	98	31	36	46
75 to <91	1	40	15	13	17
≥91	0	7	2	2	1

*LAZ, length-for-age z-score; WAZ, weight-for-age z-score; WLZ, weight-for-length z-score; WIC, Special Supplemental Nutrition Program for Women, Infants and Children; CDC, Centers for Disease Control and Prevention*.

a *Growth deceleration/faltering was defined based on the change in percentile-based categories of weight-for-length, weight-for-age and length-for-age between ≤ 6 and >7 months of age. Categories were defined at the 0.4th, 2nd, 9th, 25th, 50th, 75th, 91st, 98th, and 99.6th percentiles. Growth deceleration/faltering was defined by a: ≥1- category decrease if below the 9th percentile at 6 months, ≥2-category decrease if below the 91st percentile at 6 months, or ≥3-category decrease for any percentile value at 6 months if the successive measurement fell below the 9th percentile*.

b *Column-header N indicates the number of children with measurements in each age interval*.

Risks of WLZ, WAZ, and LAZ growth deceleration/faltering were low for children who received each type of WIC infant package at 7 months of age. Risks of WLZ, WAZ, and LAZ deceleration/faltering were significantly higher among children who received the breastfeeding WIC package relative to children who received the mixed feeding WIC package (Table 3). At 9 months of age, children who received the breastfeeding WIC package at 7 months had 44% higher risk of WLZ deceleration/faltering (RR 1.44, 95% CI 1.17–1.77), 64% higher risk of WAZ deceleration/faltering (RR 1.64, 95% CI 1.43–1.89), and 46% higher risk of LAZ deceleration/faltering (RR 1.46, 95% CI 1.24–1.71) than children who received the mixed feeding WIC package. These associations remained statistically significant and of similar magnitudes through 24 months. The risks of WLZ and WAZ growth deceleration/faltering were significantly lower among children who received the formula feeding WIC package compared to the mixed feeding WIC package. At 9 months of age, children who received the formula feeding WIC package at 7 months had 23% lower risk of WLZ deceleration/faltering (RR 0.77, 95% CI 0.65–0.93) and 31% lower risk of WAZ deceleration/faltering (RR 0.69, 95% CI 0.60–0.78) than children who received the mixed feeding WIC package. These associations remained significant and of similar magnitudes through 24 months. No significant difference in risk of LAZ deceleration/faltering was observed between children who received the formula feeding and mixed feeding WIC packages at 9, 12, or 18 months of age, but children who received the formula feeding WIC package at 7 months had 13% lower risk of LAZ deceleration/faltering at 24 months of age (RR 0.87, 95% CI 0.77, 0.99) than children who received the mixed feeding infant package.

**Table 3:** Risk ratios for growth deceleration/faltering^a^ among WIC-participating children in Southern California, 2010-2019, by WIC infant package received at 7 months (*N* = 16,255).

	**Age at growth deceleration/faltering determination**								
**Infant package at month 7**	**Ever^b^**	**9 months**		**12 months**		**18 months**		**24 Months**	
	**%**	**RR**	**95% CI**	**RR**	**95% CI**	**RR**	**95% CI**	**RR**	**95% CI**
**WLZ**									
Mixed feeding	3.53	1.00	-	1.00	-	1.00	-	1.00	-
Formula feeding	2.30	0.77	0.65, 0.93	0.72	0.62, 0.84	0.68	0.59, 0.78	0.70	0.61, 0.80
Breastfeeding	4.77	1.44	1.17, 1.77	1.46	1.22, 1.73	1.50	1.29, 1.74	1.54	1.33, 1.79
**WAZ**									
Mixed feeding	4.92	1.00	-	1.00	-	1.00	-	1.00	-
Formula feeding	2.81	0.69	0.60, 0.78	0.65	0.58, 0.74	0.64	0.57, 0.72	0.68	0.59, 0.77
Breastfeeding	6.90	1.64	1.43, 1.89	1.70	1.50, 1.93	1.78	1.58, 2.01	1.83	1.60, 2.09
**LAZ**									
Mixed feeding	4.89	1.00	-	1.00	-	1.00	-	1.00	-
Formula feeding	3.98	0.94	0.82, 1.07	0.94	0.83, 1.07	0.92	0.82, 1.04	0.87	0.77, 0.99
Breastfeeding	6.25	1.46	1.24, 1.71	1.49	1.29, 1.73	1.52	1.32, 1.74	1.48	1.28, 1.70

*LAZ, length-for-age z-score; WAZ, weight-for-age z-score; WLZ, weight-for-length z-score; mo, months; WIC, Special Supplemental Nutrition Program for Women, Infants and Children; RR, risk ratio; CI, confidence interval*.

a *Growth deceleration/faltering was defined based on the change in percentile-based categories of weight-for-length, weight-for-age and length-for-age between ≤ 6 and >7 months of age. Categories were defined at the 0.4th, 2nd, 9th, 25th, 50th, 75th, 91st, 98th, and 99.6th percentiles. Growth deceleration/faltering was defined by a: ≥1- category decrease if below the 9th percentile at 6 months, ≥2-category decrease if below the 91st percentile at 6 months, or ≥3-category decrease for any percentile value at 6 months if the successive measurement fell below the 9th percentile*.

b *Percent of children who experienced growth deceleration/faltering between 6 months of age and any subsequent measurement*.

## Discussion

Prompted by a reported caloric deficit at 7 months among mixed-fed infants participating in WIC (14), this study investigated whether receipt of the mixed feeding package at 7 months of age was associated with infant growth deceleration/faltering. Growth deceleration/faltering occurred infrequently, evident in 1.1- 3.3% of children at any age from 7 to <26 months enrolled in WIC in Southern California between 2010 and 2019. Compared to children who received the mixed feeding infant package from WIC at 7 months of age, the risks of WLZ, WAZ, and LAZ deceleration/faltering at 9, 12, 18, and 24 months of age were lower among children who received the formula feeding infant package from WIC, and higher among children who received the breastfeeding infant package from WIC. The differences were statistically significant in this large sample, but small on an absolute scale.

To our knowledge, no prior evaluation of growth deceleration/faltering by food package provided by WIC has been undertaken. Moreover, no similar study comparing the impact of infant feeding on growth deceleration/faltering among a general (i.e., not recruited for a prior medical condition) infant population was identified in the United States. However, a population-based study conducted among a cohort of all children in the catchment area of the Avon Health Authority in England identified failure to thrive between 8 weeks and 9 months of age in 4.2% of sampled children, which was more prevalent among children breastfed longer than 6 months (21). The method of identifying growth deceleration/faltering in the current study was different, but a similar pattern was observed with increased prevalence of decelerated/faltering growth among children fully breastfeeding beyond 6 months. The authors concluded that the introduction of complementary foods represents a critical period in which parents may need additional support to successfully meet the nutritional needs of their children (21). WIC food packages are accompanied by nutrition education, including information for parents on optimal complementary feeding (12, 22). Results suggest this support may be important, particularly for parents of fully breastfed infants during the complementary feeding transition. A recent review of interventions for growth faltering among infants younger than 6 months found mixed results among studies comparing the impacts of formula and breastmilk on growth among children with growth failure or other nutritional risks (23). Similar to the results in this study, another review found that formula contributed to faster and more linear growth among pre-term and low birthweight infants than breastmilk (24); however, these prior results are not directly comparable to those of the present study because the prior study did not include full term and normal birthweight infants while the present study did.

The proportion of children who experienced growth deceleration/faltering was in line with previous estimates (21); however, this study may not capture the true prevalence of decelerating/faltering growth due to sample exclusion criteria, an insufficiently specific definition of growth deceleration/faltering (17, 18), and reliance on the assumption that the growth standard used fully represents healthy growth trajectories (25, 26). The definition of growth deceleration/faltering used may also capture regression to the mean for children who were large at baseline measurements (27).

Children who received the mixed feeding package from WIC at 7 months of age had a lower risk of each type of growth deceleration/faltering at 9, 12, 18, and 24 months relative to children who received the breastfeeding package at 7 months. The reported caloric deficit among mixed-fed children from a nationally representative sample of WIC participants in 2013–2014 (14), then, does not appear to have contributed to elevated risks of WLZ, WAZ, and LAZ deceleration/faltering between 9 and 24 months of age. The reason for the higher risk of growth deceleration/faltering among children receiving the breastfeeding package at 7 months is unlikely to be due to caloric deficits, as there was no reported caloric deficit for this group from the prior study (14). Caloric intake from breastmilk in that study was estimated based on infant age, breastfeeding exclusivity, and the amount of other milk consumed (14, 28). Previous reports provide evidence that mothers engaging in breastfeeding may overestimate the amount of breastfeeding being done—particularly among those not fully breastfeeding (8), and research has identified lower caloric intake among breastfeeding infants compared to formula feeding infants (29, 30), though these differences did not contribute to elevated morbidity (30). Additional research has identified slower growth among breastfeeding infants between 6 and 18 months of age, which could help explain the observed association (31). The overestimation of the amount of breastmilk consumed, and slower growth among breastfeeding infants, may explain the higher risk of growth deceleration/faltering. On the other hand, children receiving the fully breastfeeding package were less likely to be classified into the high and very-high categories for WLZ, WAZ, and LAZ at all ages. Breastfeeding has been found to be associated with more favorable growth patterns and lower obesity risk among WIC-participating children in Los Angeles County (32–34).

Dairy products, a component of most infant formulas, are associated with linear growth, which may contribute to differences in growth patterns between breastfed, mixed-fed and formula-fed infants (35). The contribution of dairy exposure from formula to more linear growth trajectories could contribute to lower growth deceleration/faltering risk, and increased obesity risk, observed with the increased amount of formula consumed (34). Overfeeding, identified among nearly 40% of formula fed WIC participants, may also increase infant growth rates early in life and, therefore, decrease growth deceleration/faltering risk (36).

Strengths of this study include a large, well-characterized study population, with prospectively collected data for exposures and outcomes. The analysis was restricted to infants with stable growth trajectories at ≤ 6 months of age, and children below the 2nd percentile at ≤ 6 months of age were excluded from the analysis. Infants with known medical conditions which could contribute to nutritional risk and growth deceleration/faltering were also excluded from the analysis. The analysis controlled for potential confounding factors by sample restriction and statistical adjustment; however, there are also limitations. Data on infant diet and caloric intake were not available, so we were unable to evaluate the association between infant package and caloric intake; however, WIC infant package assignment has been identified as a valid proxy for infant feeding patterns (8). Children with undiagnosed underlying medical conditions which contribute to growth deceleration/faltering may have been included in the sample. The association between infant package and growth deceleration/faltering may be confounded by different exposure to dairy products, a component of most infant formulas, which are associated with linear growth (35). Because the data were administrative, potential confounding factors that may be associated with infant feeding decisions including maternal employment status were unknown. Statistically significant associations between WIC infant package and growth deceleration/faltering were observed and relative risks demonstrate moderate magnitudes of association, but absolute differences in the proportion of participants exhibiting growth deceleration/faltering were <3% for all group comparisons.

## Conclusions

Caloric intake insufficient for growth maintenance leads to growth deceleration/faltering. Nutrition assistance from WIC aims to alleviate food insecurity and provide children in low-income households access to nutritionally adequate diets that will support growth. The low rates of growth deceleration/faltering identified among all children studied highlight the critical importance of nutrition assistance among WIC participants, and demonstrate the need for the provision of education and foods to support adequate nutrition in the early months of life. Breastfeeding provides many health benefits and represents the best infant feeding practice from a public health perspective (37). Additional support, including education and individualized counseling, for breastfeeding mothers and infants during the complementary feeding transition is encouraged to promote optimal growth and minimize any risk of growth deceleration/faltering.

## Data Availability Statement

The data analyzed in this study is subject to the following licenses/restrictions: the authors used confidential administrative data for this article, and are unable to make these data publicly available due to a memorandum of understanding with the California Department of Public Health WIC Division. Requests to access these datasets should be directed to the Data Mining Project, datamining@phfewic.org.

## Ethics Statement

The studies involving human participants were reviewed and approved by University of California Los Angeles Institutional Review Board. Written informed consent for participation was not required for this study in accordance with the national legislation and the institutional requirements.

## Author Contributions

MC, CA, and SW designed the study. MC, CC, and CA conducted the research and statistical analysis. SW provided essential materials. CA drafted the manuscript and CC, MC, MW, and SW edited the manuscript. All authors were involved in the interpretation of the results, approved the final version for submission, and agree to be responsible for its contents.

## Funding

Funding from a research partnership with First 5 LA (Contract 07030) supported the analysis time of CA.

## Conflict of Interest

The authors declare that the research was conducted in the absence of any commercial or financial relationships that could be construed as a potential conflict of interest.

## Publisher's Note

All claims expressed in this article are solely those of the authors and do not necessarily represent those of their affiliated organizations, or those of the publisher, the editors and the reviewers. Any product that may be evaluated in this article, or claim that may be made by its manufacturer, is not guaranteed or endorsed by the publisher.

## Supplementary Material

Supplementary Figure 1 Flow chart for WIC-participating children served by PHFE WIC between 2010 and 2019 included in the analytic sample.

Supplementary Table 1 Parameter estimates from modified Poisson regression models for weight-for-length, weight-for-age, and length-for-age growth deceleration/faltering.
