# Supplementary material for: Mixed Infant Feeding Is Not Associated With Increased Risk of Decelerated Growth Among WIC-Participating Children in Southern California
Source: Front Nutr. 2021 Oct 28;8:723501. doi: 10.3389/fnut.2021.723501 (PMC8581497; doi:10.3389/fnut.2021.723501)
Supplement: Supplementary Table 1 — Parameter estimates from modified Poisson regression models for weight-for-length, weight-for-age, and length-for-age growth deceleration/faltering. [file Table_1.DOCX]

|  |  | Weight-for-Length | | Weight-for-Age | | Length-for-Age | |
| --- | --- | --- | --- | --- | --- | --- | --- |
| Variable | | Est (SE) | p | Est (SE) | p | Est (SE) | p |
| Intercept | | -5.54 (0.34) | <.01 | -4.55 (0.29) | <.01 | -6.16 (0.33) | <.01 |
| WIC package (7m) | |  |  |  |  |  |  |
|  | Breastfeeding | 0.21 (0.24) | 0.38 | 0.01 (0.20) | 0.95 | -0.23 (0.17) | 0.18 |
|  | Formula feeding | 0.35 (0.28) | 0.21 | 0.35 (0.22) | 0.11 | 0.18 (0.20) | 0.36 |
|  | Mixed feeding | 0.00 (0.00) | ref | 0.00 (0.00) | ref | 0.00 (0.00) | ref |
| Age, linear (y) | | 5.65 (0.29) | <.01 | 7.06 (0.27) | <.01 | 4.75 (0.22) | <.01 |
| Age, linear* WIC package | |  |  |  |  |  |  |
|  | Breastfeeding | -0.88 (0.35) | 0.01 | -0.76 (0.34) | 0.02 | 0.35 (0.28) | 0.21 |
|  | Formula feeding | -0.02 (0.41) | 0.97 | 0.25 (0.37) | 0.51 | 0.39 (0.33) | 0.23 |
|  | Mixed feeding | 0.00 (0.00) | ref | 0.00 (0.00) | ref | 0.00 (0.00) | ref |
| Age, quadratic (y) | | -2.21 (0.13) | <.01 | -3.14 (0.14) | <.01 | -1.94 (0.11) | <.01 |
| Age, quadratic* WIC package | |  |  |  |  |  |  |
|  | Breastfeeding | 0.38 (0.16) | 0.02 | 0.37 (0.18) | 0.04 | -0.23 (0.14) | 0.11 |
|  | Formula feeding | 0.05 (0.19) | 0.80 | -0.07 (0.19) | 0.70 | -0.19 (0.17) | 0.25 |
|  | Mixed feeding | 0.00 (0.00) | ref | 0.00 (0.00) | ref | 0.00 (0.00) | ref |
| Age, cubic (y) | | 0.26 (0.02) | <.01 | 0.39 (0.02) | <.01 | 0.23 (0.02) | <.01 |
| Age, cubic* WIC package | |  |  |  |  |  |  |
|  | Breastfeeding | -0.04 (0.02) | 0.04 | -0.04 (0.02) | 0.07 | 0.04 (0.02) | 0.07 |
|  | Formula feeding | -0.01 (0.03) | 0.69 | 0.01 (0.03) | 0.80 | 0.02 (0.02) | 0.28 |
|  | Mixed feeding | 0.00 (0.00) | ref | 0.00 (0.00) | ref | 0.00 (0.00) | ref |
| Race/ethnicity | |  |  |  |  |  |  |
|  | Asian | 0.67 (0.26) | 0.01 | 0.52 (0.22) | 0.02 | 0.98 (0.27) | <.01 |
|  | Black | 0.50 (0.27) | 0.07 | -0.12 (0.24) | 0.61 | 0.10 (0.28) | 0.73 |
|  | Hispanic | 0.19 (0.25) | 0.44 | -0.07 (0.20) | 0.73 | 0.58 (0.24) | 0.01 |
|  | Other | 0.04 (0.30) | 0.89 | -0.21 (0.26) | 0.43 | 0.38 (0.30) | 0.20 |
|  | White | 0.00 (0.00) | ref | 0.00 (0.00) | ref | 0.00 (0.00) | ref |
| Maternal education | |  |  |  |  |  |  |
|  | < High school | 0.17 (0.08) | 0.03 | 0.18 (0.07) | 0.01 | 0.17 (0.07) | 0.02 |
|  | High school degree | 0.06 (0.07) | 0.44 | 0.06 (0.07) | 0.33 | 0.06 (0.07) | 0.40 |
|  | >High school | 0.00 (0.00) | ref | 0.00 (0.00) | ref | 0.00 (0.00) | ref |
| Male |  | 0.21 (0.05) | <.01 | 0.11 (0.05) | 0.02 | -0.28 (0.05) | <.01 |
| Maternal language | |  |  |  |  |  |  |
|  | Spanish | -0.31 (0.13) | 0.02 | -0.18 (0.12) | 0.14 | 0.15 (0.14) | 0.31 |
|  | Other | -0.30 (0.15) | 0.04 | 0.06 (0.13) | 0.63 | 0.32 (0.15) | 0.03 |
|  | English | 0.00 (0.00) | ref | 0.00 (0.00) | ref | 0.00 (0.00) | ref |
| Income <100% FPL | | -0.02 (0.04) | 0.55 | 0.00 (0.03) | 0.88 | -0.03 (0.03) | 0.34 |
| Initial z-score | | -0.29 (0.01) | <.01 | -0.45 (0.01) | <.01 | -0.14 (0.01) | <.01 |
| QIC | | 31779.9 | - | 34825.8 | - | 35452.2 | - |

**Supplemental Table**. Parameter estimates from modified Poisson regression models for weight-for-length, weight-for-age, and length-for-age growth deceleration/faltering.

QIC=quasi-likelihood information criterion; FPL=federal poverty level; WIC=Special Supplemental Nutrition Program for Women, Infants and Children; m=months; y=years; Est=parameter estimate; SE=standard error; ref=reference.
